# Lost in translation: Assessing the readability of online information on community pharmacy services

**DOI:** 10.1177/17151635251332612

**Published:** 2025-05-22

**Authors:** Bobbi Morrison, Todd A. Boyle, Sammy Johnson

**Affiliations:** Gerald Schwartz School of Business, St. Francis Xavier University, Antigonish, NS; Gerald Schwartz School of Business, St. Francis Xavier University, Antigonish, NS; Gerald Schwartz School of Business, St. Francis Xavier University, Antigonish, NS

## Abstract

**Background:**

The World Health Organization’s right to health underscores the need for accessible, acceptable, and quality health services. Given that most Canadians use the Internet for health information, the readability of online pharmacy services information is crucial for accessibility.

**Methods:**

This study assessed the readability of online information about pharmacy services from Canadian provincial pharmacy regulatory authorities (PRAs) and community pharmacy banners. Public-facing website content was evaluated using various readability tests. Scores were compared to recommended reading grade levels by health organizations, and differences between PRA and community pharmacy banner websites were analyzed.

**Results:**

Website content from 9 PRAs and 10 community pharmacy banners was analyzed in June 2024. Average readability scores exceeded the recommended eighth-grade level, with summary scores ranging from 8.45 to 15.28. International English Language Testing System scores for all websites also surpassed reading benchmarks necessary for Canadian immigration. Mann–Whitney *U* tests indicated statistically significant differences between PRA and community pharmacy banner websites, with the latter being more readable.

**Discussion:**

The results suggest that both PRAs and community pharmacy banners provide information at an advanced reading level, hindering accessibility. This aligns with other research indicating that online health information is often too complex for the general public. Improving readability, particularly for new Canadians, is essential for better accessibility.

**Conclusion:**

Public health information on PRA and community pharmacy banner websites generally exceeds the recommended readability level, limiting accessibility. Implementing readability assessments and plain-language standards can enhance the accessibility and engagement of online health information.

## Background

The World Health Organization’s right to health emphasizes the importance of ensuring that everyone has access to the health services they need, when and where they need them, without suffering financial hardship.^1^ The right to health includes elements of availability, accessibility, acceptability, and quality of health services (AAAQ), and this framework^2^ may be used to understand how health organizations and programs are achieving the right to health.^3,4^ As community pharmacists are important primary care providers whose roles have become increasingly visible since the COVID-19 pandemic,^5-9^ a similar human rights-based analysis of health services in the context of community pharmacies may also be applied.^10^

**Knowledge into Practice**

- Online health information often exceeds recommended readability levels, making it difficult for many to understand.
- This study shows that information about community pharmacy services provided by both pharmacies and pharmacy regulatory authorities exceeds recommended reading levels, with pharmacy regulatory authorities providing less readable information than community pharmacy banners.
- Pharmacies and regulators should integrate readability assessments into content development, use plain language, and simplify content to improve accessibility, especially for newcomers.

Accessibility, within the AAAQ framework, encompasses physical, financial, administrative, social, and information dimensions.^2^ Information accessibility ensures that individuals can seek, receive, and understand health information, which is vital for informed decision-making. One way that information about community pharmacies, the roles of pharmacists, and pharmacy services is communicated to the public is through the websites of community pharmacies and provincial pharmacy regulatory authorities (PRAs). Considering that 94% of Canadians accessed the Internet in 2024^11^ and most Canadians (69%) use the Internet to access health information,^12^ websites are important vehicles to communicate with the public about pharmacy services.

The readability of a website is fundamental to its accessibility.^13^ Many Canadians read at a low proficiency level, even those with a university education.^14^ In the most recent Programme for the International Assessment of Adult Competencies (PIAAC), just over 16% of Canadian adults were found to score at the lowest 2 reading proficiency levels, with 12.6% possessing the skills to read information of limited complexity in the absence of other distracting information (level 1), and a further 3.8% were unable to demonstrate literacy skills at that level (below level 1).^15^ Reading proficiency levels are even lower for some recent and established immigrants to Canada, with 25% of individuals in these groups observed to fall within the lowest 2 reading levels on the PIAAC (level 1 and below level 1).^16^ As immigration rates have surged in recent years and are forecast to increase further,^17^ attending to the readability of online information about pharmacy services becomes increasingly important to ensure information accessibility for all.

**Mise En Pratique Des Connaissances**

- L’information sur la santé en ligne dépasse souvent les niveaux de lisibilité recommandés, ce qui la rend difficile à comprendre pour de nombreuses personnes.
- Cette étude montre que l’information sur les services de pharmacie communautaire fournis par les pharmacies et les organismes de réglementation des pharmacies dépasse les niveaux de lecture recommandés, les organismes de réglementation des pharmacies fournissant des informations moins lisibles que les bannières de pharmacies communautaires.
- Les pharmacies et les organismes de réglementation devraient intégrer des évaluations de la lisibilité dans l’élaboration de leur contenu, utiliser un langage simple et simplifier le contenu pour en améliorer l’accessibilité, notamment pour les nouveaux arrivants.

To contribute to information accessibility, information about pharmacy services must be readable by all members of the public, including those with lower levels of reading proficiency and those whose first language may not be 1 of Canada’s official languages (e.g., immigrants). Existing readability research in the context of pharmacy has tended to focus on medication-related information, including medication inserts,^18-20^ information about medication side effects,^21^ online medication information provided by community pharmacies,^22^ patient information leaflets about COVID-19 vaccines,^23^ and patient education materials about deprescribing.^24^ While this research has addressed key aspects of information accessibility in pharmacies, it overlooks the broader range of pharmacy services that are becoming progressively relevant as community pharmacists’ scope of practice expands. As pharmacists take on more responsibilities, it becomes increasingly important that the information about these services is accessible to the public. Despite the growing importance of these services, there do not appear to be any studies that specifically examine the readability of online information about pharmacy services. This research aims to fill this gap.

## Research objectives

The objectives of this study were 1) to assess the readability of information about pharmacy services provided online to the public and 2) to compare the readability of information about pharmacy services provided online to the public by pharmacy chains with that provided by provincial PRAs.

## Methods

Website content from public-facing information presented on pharmacy-focused webpages was first converted into plain text using Textise (textise.net). For this study, pharmacy-focused webpages included Canadian English language provincial PRAs, individual community pharmacy banner websites, and websites that represent a group of banners based on common ownership (360Health Pharmacy & Wellness for Sobeys-owned banners). In cases where the webpage prevented Textise access, the webpage text was manually copied into an MS Word document and saved as plain text.

On PRA sites, information in “For the Public” or “Public Protection” tabs was collected. On pharmacy sites, information pertaining to “Pharmacy Services” was collected. All connected html pages were also converted to plain text using Textise. Any links to Portable Document Format (PDF) files or external sites were not converted. Text files were manually cleaned to delete any site map lists or website footers, including copyright statements. When present, fillable form content (name, location) was deleted, as were links to print, share, or connect with social media. Alt text for images was retained when included within page content. Alt text included as part of a website footer (e.g., PRA or pharmacy logo) was removed with the footer info. All website data were captured between June 4 and 6, 2024.

Various readability test tools were used to assess ease in reading and understanding the webpage text. These tests apply specific formulas to sections of text to assess their reading complexity. Various characteristics of the text, such as average sentence length (ASL), average number of syllables per word (ASW), and average number of characters per word (ACW), were used to assign a readability score that may be based on a 100-point scale (Flesch–Kincaid readability tests) or the expected reading competency within the US school system (Flesch–Kincaid grade level). The latter scale assesses reading competency expected at specific grade levels and not educational attainment based on years in school. Regardless of scoring approach, these tests have the overall goal of identifying the reading proficiency needed to comprehend specific passages of text and allow content creators to assess whether the readability of the text is appropriate for the intended audience.

Readability tests using the plain text files of community pharmacy banners and PRA websites were completed using the online readability assessment tool, Readable (readable.com). For this study, 6 common tests that measure readability using the US grade school level^25^ were selected. A summary of the specific tests^25^ is presented in Table 1. Among these, the Simple Measure of Gobbledygook (SMOG) is preferred for health information.^26-28^ Details of the formula used for each test are available at Readable.^25^

**Table 1:** Readability tests

Test	Test summary
Flesch–Kincaid grade level	Estimates the reading grade level of a text by analyzing average sentence length and word complexity. The scores it generates correspond to US grade levels.
Gunning Fog Index	Measures text complexity by estimating the education level needed to understand the text. It considers ASL and the percentage of complex words (words with 3 or more syllables).
Coleman–Liau Index	Assesses readability by analyzing the average number of letters per 100 words and the average number of sentences per 100 words.
Simple Measure of Gobbledygook (SMOG)	Estimates the education level required to understand text by counting the number of polysyllabic words in a sample of 30 sentences.
Automated Readability Index (ARI)	Evaluates readability based on ASL and the average number of characters per word (ACW). It is useful for technical writing.
Ford, Caylor, and Sticht (FORCAST)	Designed for technical documents, it assesses readability based on the number of monosyllabic words in a 150-word sample.

ASL, average sentence length.

Different health organizations recommend various grade-level targets for materials aimed at the public. The National Institutes of Health (NIH) recommend presenting materials below a grade 8 level,^29-31^ the American Medical Association (AMA) recommends grade 6 or lower,^31,32^ the Centers for Disease Control and Prevention recommend grade levels of 5 to 8,^33^ and the Public Health Association of Canada recommends grade levels of 4 to 6.^34,35^ For this study, the top of the recommended range (grade 8) has been used to analyze data.

Recognizing that all of the tests in Table 1 deploy different formulas and measure slightly different aspects of readability, a summary score was also used for this study. The score, calculated after the readability scores in Table 1 were generated, is the average of all 6 readability test scores for each community pharmacy banner and PRA. Analyses of basic statistics (mean, median) and differences between groups (PRA, community pharmacy banner) using non-parametric statistics (Mann–Whitney *U*) were performed.

The International English Language Testing System (IELTS) is a standardized test used to assess the English language skills of non-native English speakers. Specifically, the test assesses English language ability along 4 criteria: reading, writing, listening, and speaking skills. IELTS scores range from 1 (non-user) to 9 (expert user). Scores are assigned in 0.5 increments to each of the 4 criteria, with a total score calculated as the average of all 4 criteria. The test is commonly used to assess English proficiency for educational, employment, and immigration purposes. Variations of the test exist depending upon the intended purpose, including academic (higher education, professional registration) and general training (immigration, general training programs) versions.^36^

The IELTS reading skill level appropriate for newcomers to Canada depends on the specific immigration program applied to, with benchmarks of 3.5 (Federal Skilled Trades Program),^37,38^ 4 (Interim Pathway for Caregivers Program, Start-Up Visa Program),^38-40^ and 6 (Federal Skilled Worker Program, Federal Internships for Newcomers Program)^37,38,41^ commonly used. This study captured the IELTS reading score for each of the PRAs and community pharmacy banner sites and compared the scores to these common benchmarks.

Ethics review was not required for this study, given that only text from publicly available webpages was assessed for readability.

## Results

A total of 9 PRAs and 10 community pharmacy banner websites were reviewed. The text reviewed for each site contained an average of 381 paragraphs, 501 sentences, 11 words per sentence, and 175 long (20–29 syllables)^42^ and 101 very long (30 syllables)^42^ sentences. Results of the data analysis indicate that most PRAs and community pharmacy banners received test scores well above the eighth-grade reading level benchmark. The average scores across the various tests (summary score) ranged from a low of 8.45 to a high of 15.28. All webpages scored poorly on the SMOG test with reading levels ranging from a low of 9.85 to a high of 16.72, all well above the NIH-recommended level of 8 or below. The individual test scores for each PRA and community pharmacy banner are presented in Table 2.

**Table 2:** Individual test scores

	Summary score	SMOG	Flesch–Kin.	Gunning	Cole–Liau	ARI	FORCAST
PRA							
AB	15.28	16.72	14.11	17.75	16.01	15.04	12.04
BC	11.72	12.52	9.71	11.13	14.66	10.72	11.59
MB	12.16	12.93	10.25	12.23	14.69	11.18	11.65
NB	11.82	12.43	10.16	10.04	15.35	11.12	11.81
NL	11.93	12.70	9.69	12.63	14.54	10.35	11.69
NS	12.20	13.76	10.46	12.80	13.57	11.13	11.46
ON	12.03	12.88	10.45	12.25	14.32	10.41	11.85
PE	12.00	12.94	9.37	11.92	14.85	10.88	12.05
SK	12.21	13.31	10.73	12.23	14.17	10.94	11.90
Banner							
Brunet	10.38	11.23	8.50	11.05	11.92	8.22	11.36
Costco	10.11	11.05	8.47	8.84	12.37	8.16	11.77
Guardian	11.09	12.08	9.35	11.79	12.82	9.00	11.50
Loblaws	11.09	11.44	8.60	10.47	14.38	10.40	11.28
PharmaChoice	10.20	11.13	8.27	10.04	12.08	8.54	11.11
Pharmasave	10.44	10.78	8.84	8.73	13.44	9.01	11.86
Shoppers	8.45	9.85	6.34	8.38	9.71	6.05	10.35
360HPW	9.51	10.25	7.60	9.44	11.19	7.24	11.37
Uniprix	10.91	11.89	9.17	11.65	12.59	8.88	11.27
Walmart	11.63	11.45	10.56	10.04	14.87	10.04	12.83

360HPW, 360Health Pharmacy & Wellness; AB, Alberta; ARI, Automated Readability Index; BC, British Columbia; Cole–Liau, Coleman–Liau test; Flesch–Kin., Flesch–Kincaid grade level; FORCAST, Ford, Caylor, and Sticht; MB, Manitoba; NB, New Brunswick; NL, Newfoundland and Labrador; NS, Nova Scotia; ON, Ontario; PE, Prince Edward Island; PRA, pharmacy regulatory authority; SK, Saskatchewan; SMOG, Simple Measure of Gobbledygook.

To further illustrate the differences in readability identified in Table 2, Table 3 presents deidentified examples of text with high and low readability scores, drawn from descriptions of pharmacists’ roles on PRA websites.

**Table 3:** Examples of higher vs lower readability

Example of higher readability score (poorer readability)	Example of lower readability score (better readability)
Your pharmacist is your drug therapy specialist and helps support you in reaching your health goals by: • Assessing your health and drug therapy needs by considering your health history, current health status, current drug therapy, diagnostic and laboratory test results, allergies, lifestyle habits, and other relevant information. They make evidence-informed decisions when performing authorized activities, including dispensing, prescribing, and administering drugs, vaccines, or other alternatives (including by injection) to treat or prevent disease, or to maintain your health. • Working with you to develop your health plan, which may include drug and non-drug alternatives. As a key member of your health team, they consider your health status, health goals, and personal preferences when assessing your overall health needs. They consult, collaborate with, and advise other health and community service providers to ensure that your care is integrated and coordinated. • Educating and supporting you to use your treatments properly. Together, you and your pharmacist monitor your health and response to your health plan. Your pharmacist may adjust your drug treatments to support reaching your health goals and minimize the impact of undesired effects. • Coaching you about healthy living (e.g., nutrition, exercise, smoking cessation, substance misuse and abuse, and sexual health). They offer screening programs to identify personal health risks, and immunization services to protect you from disease. • Directing your pharmacy team and ensuring the drugs and services you receive are safe and of high quality, and delivered in a professional and ethical manner. When your team does not include a pharmacy technician, your pharmacist takes responsibility for all roles that a pharmacy technician may provide.	Pharmacists are regulated health professionals with university-level education in pharmacy. They must: • Complete training in dispensing prescription and non-prescription medications, drug therapy, understanding and managing drug interactions and side effects, and patient education and counselling • Understand all laws governing pharmaceuticals, pharmacist/technician roles, and pharmacy operations • Successfully complete national and provincial exams demonstrating competence in pharmacy practice and applicable pharmacy laws • Meet continuing education and competence requirements • Uphold the standards of practice set out in the National Association of Pharmacy Regulatory Authorities (NAPRA) Model Standards of Practice for Canadian Pharmacists • Follow the [province] College of Pharmacy Code of Ethics Pharmacists are responsible for the aspects of pharmacy practice requiring clinical judgment such as assessing the appropriateness of drug therapy, managing drug interactions and side effects, and educating patients on medication.

PRAs and community pharmacy banners on average scored the best (lowest scores) on the Flesch–Kincaid test and worst (highest scores) on the Coleman–Liau test. A summary of the average scores for each test is presented in Table 4.

**Table 4:** Readability score summary

	*N*	Mean score	Median score	Minimum	Maximum	Standard deviation
Summary score	19	11.32	11.63	8.45	15.28	1.41
SMOG	19	12.18	12.08	9.85	16.72	1.53
Flesch–Kincaid	19	9.51	9.37	6.34	14.11	1.59
Gunning	19	11.23	11.13	8.38	17.75	2.10
Coleman–Liau	19	13.55	14.17	9.71	16.01	1.60
ARI	19	9.86	10.35	6.05	15.04	1.93
FORCAST	19	11.62	11.65	10.35	12.83	.49

ARI, Automated Readability Index; FORCAST, Ford, Caylor, and Sticht; SMOG, Simple Measure of Gobbledygook.

Non-parametric tests were used to explore any differences in scores between the pharmacy regulatory authority and community pharmacy banner groups. Specifically, the Mann–Whitney *U* test was used to explore any differences in the summary test score between these 2 groups. Results of the Mann–Whitney *U* test indicate a significant difference in test scores between pharmacy regulatory authorities (median = 12.03) and community pharmacy banners (median = 10.41), *z* = –3.67, *P* < .001, with community pharmacy banner webpages scoring a lower overall readability score and therefore possessing easier to comprehend webpages when compared to those of the PRAs. The IELTS reading scores for all the webpages also scored poorly with reading levels at 8+ (very good and above) and well above the immigration benchmarks of 3.5 (extremely limited to limited), 4 (limited), and 6 (competent).

## Discussion

Pharmacy regulatory authorities play a crucial role in protecting the public. To help meet this mandate, the information communicated to the public must be accessible to those with varying levels of literacy and understanding of Canada’s official languages. Similarly, community pharmacy banners serve the health needs of a wide variety of patients with varying levels of literacy, as well as non-native English speakers. Yet, our findings suggest that public-facing communication from these entities is presented at a level that may be too high for all members of the public to be able to read. This is consistent with other research studies. For example, in their systematic review of 157 studies examining public-facing health information on websites between 2007 and 2017, Daraz et al. (2018)^31^ revealed that websites in Canada and the United States present health information at a level too advanced for the general public. Numerous recent studies from around the world have also identified that the readability of health information online is written at a level too advanced for the public to read.^33,43-47^ However, research also shows that health regulators have been successful communicating complex health information at a level appropriate to the literacy levels of the public, demonstrating that achieving accessibility in health communication is feasible.^23^

In communicating with the public, PRAs and community pharmacies must also be mindful of the literacy levels of new Canadians. Newcomers to Canada experience challenges navigating the Canadian health care system,^48,49^ and language barriers and access to information have persistently been identified as major access barriers.^48-52^ Our findings reveal that the ability to read information about pharmacy services from both PRAs’ and community pharmacies’ websites requires an IELTS score far exceeding that required by all immigration programs in the country,^37-41,53-57^ meaning that the content may not be readily accessible to new Canadians. This suggests that there are clear opportunities for PRAs and community pharmacies to simplify their communication to help minimize the language barriers experienced by immigrants in accessing the health care system in Canada.

Overall, the findings highlight the need to improve the readability of public- and patient-focused information and for Canadian PRAs and pharmacy banners to perform more thorough assessments of such information. Indeed, the adoption of readability calculators has helped develop more easily understood health materials,^23^ and the use of a readability tool is among the recommended standards for online patient health information.^58^ Although several readability test options are available, each with a slightly different focus, the process of assessing text readability is relatively automated, especially when reading a text document (e.g., MS Word file). This assessment can be easily performed before making information publicly available. Given their relative ease and benefits, formal readability assessments should be added to the existing quality checks (e.g., factual accuracy, grammar, and spell checks) that occur before releasing information to the public.

To enhance readability, information should be written in plain language,^34,59^ which means that “its wording, structure, and design are so clear that the intended readers can easily find what they need, understand what they find, and use that information.”^60^ Some countries, including the United States and New Zealand, have enacted plain-language laws that are applicable at the federal level.^61,62^ In Canada, plain-language standards for federal departments are currently under development,^63^ and certain regional health agencies in Canada, including Alberta Health Services, have published guidance for communicating in plain language.^64^ Given these legislative developments and the findings of this study, there is an opportunity for PRAs, once they have improved the readability of their own public-facing material, to champion the issue and establish standards of practice for readability and plain language to contribute to information accessibility for all.

The implications of poor readability, however, extend well beyond not fully understanding the information presented. For example, the public has an important role in assisting PRA oversight (complaints-driven investigations). To meaningfully engage, the public must be able to read and comprehend information about how to do so. Additionally, transparency is thought to be necessary for PRAs to demonstrate their trustworthiness to the public.^65^ However, transparency cannot exist if the information provided is beyond the reach of the public. For pharmacy banners, enhanced readability also makes good business sense. Readability scores affect online search results,^66,67^ meaning that less readable sites do not rank well in Internet searches. Consequently, the information on less readable sites becomes even less accessible, while more readable sites from competitors appear higher in search results.

Several limitations to this study also represent worthy future research opportunities. This study focused on 2 key sources of health information, specifically PRA and pharmacy banner chain websites. However, there are also many other sources of information available to patients, including printed brochures, webpages of allied health services (e.g., Health Canada website), and conversations with health care professionals (pharmacists, nurse practitioners, physicians). Future research is needed to test the readability of the web-based and printed materials produced by such groups, as well as to assess the overall level of understanding from conversations with health care professionals.

While readability tests are a good starting point, they provide a limited view of a reader’s ability to understand the website content presented to the public. Inherent weaknesses of such tests include using very basic quantitative characteristics, such as sentence, syllable, and word counts in their formulas, and do not capture how content organization or visual cues (pictures, videos), bullet points, and lists help with understanding.^59^ Future research should examine the broader aspects of information presentation, including layout, design, and user engagement strategies, to gain a more complete understanding of accessibility. Qualitative approaches such as interviews and focus groups with pharmacy patients, newcomers to Canada, and non-native English speakers would also provide valuable insights into how text is understood and help to develop best practices to enhance accessibility. Finally, the summary score calculated herein was included for informational purposes. As it is not a validated approach, its validity remains unknown. Despite such limitations, this study highlights that on the surface, both PRAs and pharmacy banners are presenting text that may not be completely understood by the intended audience. Increased awareness of the issue and embedding readability testing as a standard quality check, much like spelling and grammar, should occur to enhance readability.

## Conclusion

This study explores the readability of public health information presented by PRAs and community pharmacy banners. An analysis of 19 websites indicates concerns regarding the readability of online health information provided by PRAs and community pharmacy banners. The findings highlight that most of this information is written at a level that exceeds the recommended eighth-grade reading level, rendering it potentially inaccessible to a substantial portion of the public, including individuals with lower literacy levels and newcomers to Canada. Through increased awareness, adoption of readability assessments and plain-language standards, and use of readability tools, both PRAs and community pharmacy banners can improve the readability of their online content and enhance public engagement with various pharmacy services. ■
